# Valedictory

**Published:** 1873-11

**Authors:** J. P. Logan


### VALEDICTORY.

A change in the basis and office of publication and in the business management of the ATLANTA MEDICAL AND SURGICAL JOURNAL, affords an opportunity, upon my part, long desired and contemplated, to retire from its editorial control. While there are weighty considerations, of an obvious character, adverse to my present course connected with the pleasant relations into which I have been brought with a large number of professional gentlemen, arising out of the editorial position I have occupied, its cares, anxieties and demands upon my time, coupled with a sufficient measure of professional labor in other directions, and greatly impaired health, have made the decision imperative.

Besides, there is no longer any (even apparent) necessity for the mission of *pacification* upon which I entered in assuming the editorial control of the JOURNAL, at a time when the elements of discord were so largely in the ascendant among the profession in this State.

All that I could have hoped for, *and more*, has been attained in the way of restoration of harmony and a return to the legitimate objects and ends of the profession, for which this JOURNAL has labored, and is believed to have been somewhat instrumental in accomplishing, and to aid in which was my principal object in yielding to the urgent solicitation to again resume editorial labors which had been years ago, as it was supposed at the time, permanently laid aside.

While others have done much more than myself in other directions in sustaining the JOURNAL, my own *special* object has been the re-establishment of fraternal relations among the medical profession of Georgia. *Peace* has been restored, and with it, as I believe, a new era in medical *science* inaugurated in the State, and I can, therefore, with perfect confidence that I leave the JOURNAL in better hands, withdraw from its responsibilities.

In thus retiring, I feel it due to myself to state that while I am conscious of many shortcomings in connection with the editorial department, I have had no pecuniary interest whatever in the JOURNAL, and have had no connection with its publication, business affairs or management, and if there are any just complaints against those who have conducted it, in these particulars, they do not lie at my door.

In thus finally taking leave of medical journalism, in connection with which so many years of my life have been spent, I cannot but experience with the pleasure arising from a relief from burdensome labor, a large admixture of *regret* that I shall no longer be in communication with a host of medical friends throughout every section of the United States, who have so zealously and efficiently sustained the conductors of this Journal in their efforts to advance the interests of the medical profession, and without whose aid but little of what has been accomplished, could have been achieved. To all those who have thus aided—comprising not only a very large proportion of the most influential and intelligent medical men of this State, but many of the most prominent throughout the entire country—I desire to commend my successors and to express my gratitude, and to bid them all, in this capacity, an affectionate farewell.

J. P. Logan, M.D.

